# Promoting Health for Adolescents: An Editorial

**DOI:** 10.3390/ijerph20146336

**Published:** 2023-07-10

**Authors:** Zisis Kozlakidis

**Affiliations:** International Agency for Research on Cancer, World Health Organization, 69366 Lyon, France; kozlakidisz@who.int

## 1. Introduction

The research area of adolescent health corresponds to the summary of a wide range of scientific interests and investigations, focusing on the first years of life of an individual [1]. It has remained an area of acute scientific interest for many decades, with persistent inclusivity both in terms of the methodologies used to investigate it, as well as in the many different inter-disciplinary approaches that have been successfully implemented over several decades. Indeed, the continuing interest of the scientific community has been evident for this Special Issue by the number of published works (26), views (over 45,000) and citations (53) that have materialized to date. This Special Issue [2], entitled “Promoting Health for Adolescents”, continues the scientific path of approaching the many facets relating to adolescent health, such as physical fitness, perceptions, behavioral and emotional determinants, nutritional/dietary influences, and molecular perspectives. Additionally, as the recent pandemic has had a tremendous impact on healthcare, it would be inevitable that a Special Issue on adolescent health would be hosting some of the most recent investigations relating to the COVID-19 impact on adolescent health. The manuscripts contained within this Special Issue are briefly presented below, grouped by the area of a broad scientific field, to highlight the different investigative strands that emerged throughout the period of this Special Issue.

## 2. Physical Activity and Fitness

Health, and in particular adolescent health, are inexorably tied to the levels of physical activity and fitness; thus, a number of publications focused on this topic. Specifically, based on the 2018 Chinese National Student Physical Fitness Standard Test data, Tong et al., used a multi-scale approach to investigate the spatial heterogeneity and regional clustering of factors influencing Chinese adolescents’ physical fitness, providing a national-level view [3]. Further granularity was achieved by a complementary publication by the same group, investigating the impact of environmental and socio-economic factors on low physical fitness, identifying opportunities for future investigations on regionalized coping strategies [4]. Using qualitative methodologies, and applying a social ecological model, the factors influencing the youth physical activity in Saudi Arabia were investigated [5], providing a snapshot from a distinctly different part of the world. Two studies from South Korea compared the physical fitness level of 142 middle school students through a physical assessment and a circuit exercise program [6], culminating in the use of responses by 5268 middle- and high-school students, aiding in the identification of criteria for an adolescent circuit exercise program to be conducted as part of the physical education class [7]. Finally, the mediating effects of exercise experience and commitment as a social support mechanism for Chinese college students during the COVID-19 pandemic was described in an effective manner [8], while a Korean study investigated the changes in the structural relationship between alienation in physical education classes, school happiness, and future healthy life expectations in adolescents after the COVID-19 pandemic [9].

## 3. Emotional Health, Behaviors, and Perceptions

Physical activities, such as the ones described above, are often identified as the visible expression of emotional adolescent health. Therefore, a few publications focused on emotions and emotional health. Using cross-sectional, survey-based data from Norway between 2017–2019, the association between physical activity organized in sports clubs, non-organized physical activity, other organized leisure-time activities, and depressive symptoms among adolescents were investigated [10]. A different survey-based study in China, inclusive of cultural insights (i.e., Suzhi), investigated the underlying mechanism(s) of teacher support and adolescents’ positive academic emotions, in relation to improving overall school performance [11]. A separate investigation was performed with 516 primary school students, on pupils’ emotions by assessing the impact of cooperative project-based learning interventions in primary school, in relation to moral emotions, online empathy, anger management, and the impact of these aspects on key competencies [12]. The relationship of adolescents to technologies and emotional health was an added topic of investigation, focusing on the future time perspective relating to problematic smartphone use [13], linking behaviors to perceptions and emotions.

The concepts affecting behaviors and perceptions were investigated further by four separate studies. In particular, the prevalence and predisposing factors related to self-medication among medical and pharmacy students in Serbia, as attitudes towards conventional and complementary medicine among future healthcare professionals can impact their future pharmacotherapy practice [14]. A cross-sectional study from Saudi Arabia described the knowledge, attitude, practices and viewpoints of undergraduate university students towards self-medication [15], while a cross-sectional study on 220 primary school pupils in Greece investigated the level of renal function knowledge [16]. As part of the COVID-19-related investigations, a Korean study assessed the differences in the importance and performance of health awareness in middle school students according to the types of online physical education classes [17]. The mental health of adolescents, as a vulnerable group in public health emergencies, was negatively affected by the pandemic and the unprecedented prevention and control measures. Chen et al. utilized a study methodology (checklist) for post-traumatic stress disorder of Chinese adolescents in the closed period after the COVID-19 outbreak, highlighting the mental impact of implemented public health measures [18]. A similar focused cross-sectional study from Norway evaluated the associations between demographic characteristics, lifestyle factors and school-related conditions, and symptoms of mental health problems in upper secondary school students following the COVID-19 pandemic [19]. Finally, Wiafe et al. reviewed the impact of neighborhoods on adolescent engagement in health-risk behaviors, such as substance use and sexual activity in sub-Saharan African countries [20].

## 4. Food, Nutrition, Biological and Molecular Aspects

A third group of submitted manuscripts involved the perspectives of food and nutrition, in particular the teen-identified indicators of targeted food marketing, with a particular focus on the aggressive marketing of unhealthy foods [21]. Two reviews also focused on food: the role of maternal diet in the risk of childhood acute leukemia [22], and the second on the evaluation of the effects of tele-psychotherapy in the treatment and prevention of eating disorders in adolescents [23]. A third review provided a narrative view of the impact of early behaviors, including nutrition, on the later development of non-communicable diseases, highlighting adolescence as the key time where disease-preventive approaches can be implemented [24].

The fourth and final thematic group that emerged in the papers published as part of this Special Issue had a biological and molecular focus. This included two cross-sectional studies: one of ocular changes in adolescents with diabetes mellitus in health facilities in Ghana [25], and one on the association of salivary insulin-like growth factor (IGF) and IGF/IGFBP-3 molar ratios with cervical vertebral maturation stages from pre-adolescent to post-adolescent transition period [26]. Furthermore, a review of factors influencing abnormal brain development focused on neuropsychiatric disorders with pluripotent stem cells-derived brain organoids [27].

## 5. Conclusions

Taken together, the above provide a successful collection of research studies on adolescent health and highlight the necessary interdisciplinarity of the work required to address current scientific questions. The COVID-19 pandemic has acted as a catalyst [28], bringing forward scientific discoveries, while at the same time leaving an indelible mark on the mental health, awareness, and attitudes of adolescents. It is hoped that the manuscripts published as part of this Special Issue will continue providing the foundation for future studies in the long-term.
